# Immune Checkpoints and Innovative Therapies in Glioblastoma

**DOI:** 10.3389/fonc.2018.00464

**Published:** 2018-10-23

**Authors:** Massimo Romani, Maria Pia Pistillo, Roberta Carosio, Anna Morabito, Barbara Banelli

**Affiliations:** ^1^Laboratory of Tumor Epigenetics, IRCCS Ospedale Policlinico San Martino, Genova, Italy; ^2^Department of Health Sciences, University of Genova, Genova, Italy

**Keywords:** glioblastoma, therapy, immune checkpoint, CTLA-4, PD-1, PD-L1

## Abstract

Targeting the Immune Checkpoint molecules (ICs; CTLA-4, PD-1, PD-L1/2, and others) which provide inhibitory signals to T cells, dramatically improves survival in hard-to-treat tumors. The establishment of an immunosuppressive environment prevents endogenous immune response in glioblastoma; therefore, manipulating the host immune system seems a reasonable strategy also for this tumor. In glioma patients the accumulation of CD4^+^/CD8^+^ T cells and Treg expressing high levels of CTLA-4 and PD-1, or the high expression of PD-L1 in glioma cells correlates with WHO high grade and short survival. Few clinical studies with IC inhibitors (ICis) were completed so far. Notably, the first large-scale randomized trial (NCT 02017717) that compared PD-1 blockade and anti-VEGF, did not show an OS increase in the patients treated with anti-PD-1. Several factors could have contributed to the failure of this trial and must be considered to design further clinical studies. In particular the possibility of targeting at the same time different ICs was pre-clinically tested in an animal model were inhibitors against IDO, CTLA-4 and PD-L1 were combined and showed persistent and significant antitumor effects in glioma-bearing mice. It is reasonable to hypothesize that the immunological characterization of the tumor in terms of type and level of expressed IC molecules on the tumor and TIL may be useful to design the optimal ICi combination for a given subset of tumor to overcome the immunosuppressive milieu of glioblastoma and to efficiently target a tumor with such high cellular complexity.

## Introduction

Since the discovery in 2005 of the clinical utility of Temozolomide in glioblastoma (GBM) patients (1), no other cytotoxic drug was added in the standard treatment protocols. In the meantime our knowledge on the molecular mechanisms deranged in GBM has had impressive advancements and the possibility of targeting these pathways has been extensively exploited in the hope to improve the current standard of care (2–4). Differently from many other tumors, in GBM the promises of molecularly targeted therapies against oncogenic alterations did not meet success in phase I/II and III trials even though they were highly promising in preclinical models; thereafter they have limited clinical utilization (5). The lack of success of targeted therapies and the limited activity of standard cytotoxic treatments in GBM, reside in the cellular complexity and clonal evolution of this tumor (6, 7). Moreover, many molecules that display strong antitumor activity *in vitro* against glioma cells and that are utilized for the therapy of other tumors, are ineffective *in vivo* because they cannot pass through the Blood Brain Barrier (BBB), or because of drug efflux, intrinsic or rapidly developing drug resistance and, last but not least, the presence of a pool of cancer cells with stemness characteristics (7).

In the recent years, targeting the so-called Immune Checkpoint molecules (ICs) which provide inhibitory signals to T cells, has offered new exciting treatment opportunities in cancer (8). Inhibition of autoreactive CD8^+^ T-cells through ICs is a physiological mechanism to prevent autoimmunity; on the other end, this mechanism inhibits the immune response against aberrant cancer cells. Differently from conventional cytotoxic or from targeted therapies that are aimed at the cancer cells, the therapies that involve the modulation of ICs attempt to redirect the function of the immune system to elicit cancer cell death. Several checkpoint molecules capable to shut down the response against neo-antigens are present on T cells as well as on tumor cells. These molecules are at the center of regulatory networks that result in immunosuppression. Antibodies against the “classic” IC molecules (CTLA-4, PD-1, PD-L1, and PD-L2) are considered the “first generation” IC inhibitors (ICis) that interfere with the immune escape of tumor cells, followed by second and third generations ICis targeting other immunoregulatory molecules and pathways (9, 10).

Immune checkpoints inhibition dramatically improved survival in hard-to-treat tumors like lung cancer and melanoma so that the therapy with IC inhibitors (ICis) has entered in the standard clinical practice for these tumors whereas clinical trials have been launched for many other tumors (8, 10).

## Blood-brain barrier, immunological mechanisms and immune checkpoints interplay in glioblastoma

**Figure 1:**
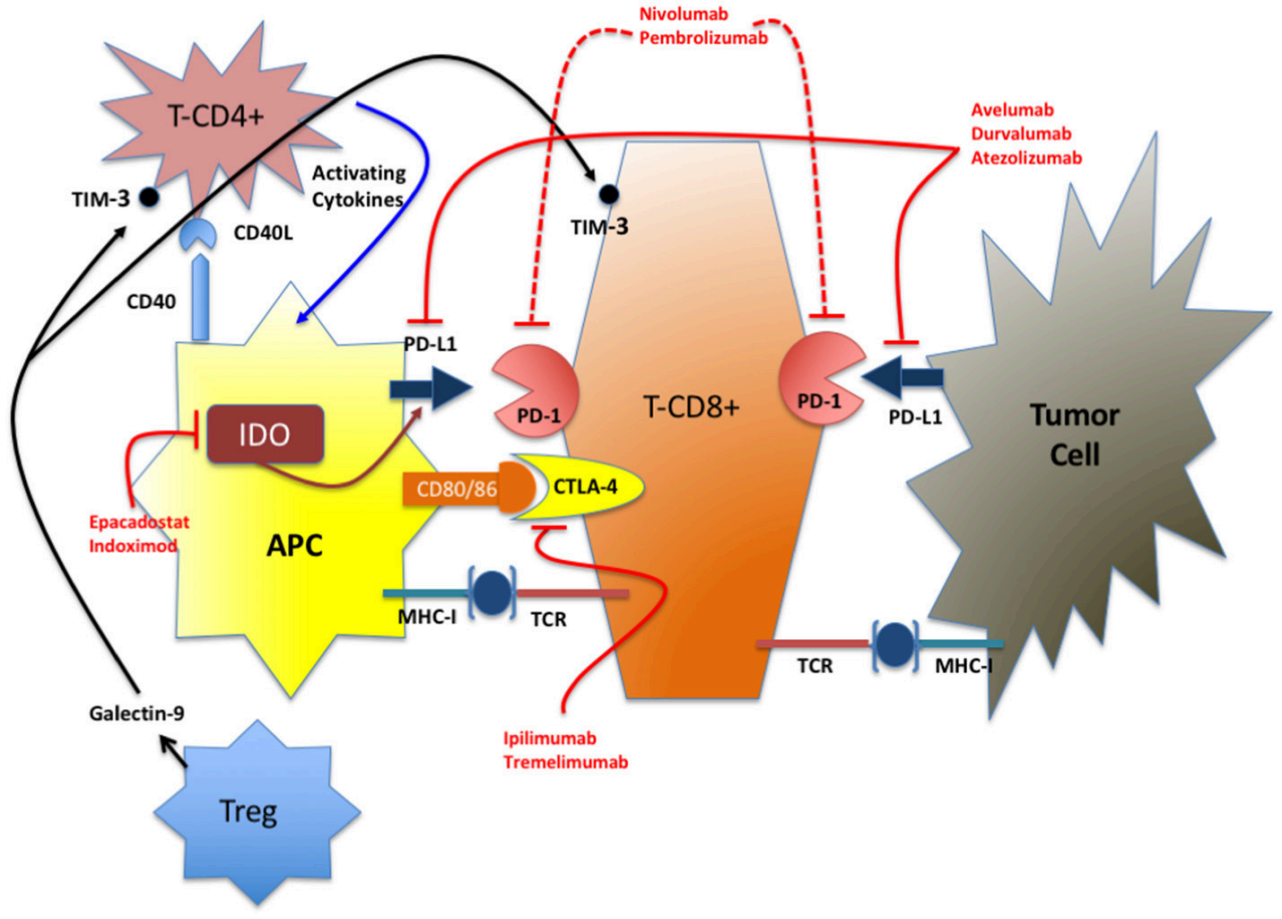
Simplified representation of the IC network. In red are indicated the FDA-approved drugs and the IDO inhibitors in advanced stage of clinical test (phase III). TIM-3 inhibitors are at an early stage of development for clinical use (phase I).

For many years the CNS has been considered as an immune-privileged compartment with the BBB responsible to maintain a constant brain microenvironment from metabolic insults and, at the same time, physically blocking or actively favoring the transport of bioactive molecules. During the development of glioma, the integrity of the BBB is preserved up to a tumor size of ~2 mm^3^; above that, the angiogenetic pressure elicited by GBM releases the tight and adherent junctions between the cerebral endothelial cells allowing the passage of molecules up to 12 nm (11, 12). With further tumor growth the BBB becomes freely permeable to larger molecules. Nevertheless, tumor cells in niches at the boundary of the surgically excised tumor remain protected by the BBB reducing the efficacy of the treatment. Beside immune cells (13), several cell types in the brain (microglia, astrocytes) can act as antigen-presenting cells and elicit immune response against the tumor. This mechanism is aided by the permeability of the damaged BBB that enables the passage of tumor antigens outside the brain (14–16). Similarly to other tumors, GBM is associated with significant immunosuppression particularly in the T-cell compartment (17) because of the combined effect of steroid/cytotoxic treatment, the downregulation of MHC-I antigens and of the secretion of immunosuppressive cytokines. Moreover, also in glioma, the correct maintenance of the physiological status of immunological tolerance and response is mediated by the coordinate interplay of many actors, including IC molecules, and different cell types as summarized below and in Figure 1.

### CTLA-4

CTLA-4 (CD152) was the first immunoregulatory molecule to be targeted for therapeutic purposes utilizing the humanized antibody Ipilimumab approved by FDA and EMA in 2011, initially for melanoma (18), soon followed by Tremelimumab for mesothelioma (19, 20). CTLA-4, expressed on T-cells (activated and regulatory), interacts with its ligands CD80 and CD86 on APCs to inhibit co-stimulators T-cell pathways (21). In GB the expression of CTLA-4 on CD4^+^ and CD8^+^ cells is strongly inversely correlated with outcome (22).

### PD-1/PD-L1

PD-1 on T cells and its ligand PD-L1 on APC and tumor cells are the most important immunosuppressive molecules so far identified. Their interaction leads to the suppression of early T-cell activation, abolishing their cytotoxic activity and interferes with the production of inflammatory cytokines (23, 24). Two PD-1 suppressive Ab were licensed in 2014 (Nivolumab) and in 2016 (Pembrolizumab) and two anti-PD-L1 Ab: Atezolizumab in 2016 and Avelumab in 2017. Their original indications were rapidly extended to other tumors and many clinical trials with newer molecules are ongoing (8, 25, 26). The expression of PD-L1 on glioma cells has been documented as well as that of PD-1 on tumor infiltrating lymphocytes (TIL). The functional and clinical implications of PD-1/PD-L1 expression in GBM are still unclear. Indeed, no correlation between PD-L1 expression and overall survival was seen in two cohorts (27, 28). On the other hand, in another study, PD-L1 staining and PD-1/PD-L1 expression were associated with decreased survival (29). The absence of standardized experimental parameters, of defined cut-off values and the heterogeneity of the cohorts might explain these contrasting findings. Interestingly PD-L1 expression is directly correlated with WHO grade and within Grade IV tumors, PD-L1 expression is significantly higher in IDH1/2 wt tumors compared to IDH1/2 mutated or hyper methylated GBM (27). Overall, the expression of PD-L1 is linked to well-known negative prognostic indicators in GBM and its effect on survival must be examined in homogeneous cohorts.

### Tim-3

Tim 3 is a molecule expressed by CD4+ and CD8+ T cells that, similarly to PD-1, is involved in immune suppression and promotes tumor escape through the exhaustion of T cells (30). A large proportion of TILs in GBM and other tumors is composed by T cells not capable of cytokine secretion and not exerting their physiological function. In GBM the overexpression of TIM-3 is associated with higher malignancy (higher grade, lower Karnofsky score, and IDHwt) and is thus considered a strong negative prognostic indicator (31, 32).

### IDO

Although IDO is not a classical immune checkpoint molecule and lacks receptorial capacity, it is included in this functional class because of its suppressive properties on T-cell activation and NK cell function (33). Similarly to TIM-3, IDO overexpression is linked to poorer outcome in GBM patients (34) and targeting IDO with Epacadostat or Indoximod (35), was a successful experimental strategy in *in vivo* models (36).

## Genetic and epigenetic factors determine the functionality of IC molecules

Targeting IC molecules with blocking antibodies alone or in combination with other ICi therapies, or with standard chemotherapy has revolutionized the therapeutic approach to lung cancer and other hard to treat tumors. Nevertheless, along with very favorable response rate, other patients are unresponsive to the therapy or show life-threatening side effects. Predicting the response and the appearance of major side effects during treatment is a major health issue. Limiting certain therapies to patients likely to respond and strict monitoring of patients at risk have several ethical implications and could enable the Public Health Systems to offer the best available therapy to the patients who could really benefit from it. However, reliable biomarkers predicting response or adverse reactions to ICi therapy are not yet available.

Single nucleotide variations (SNVs) of IC genes can affect the expression levels of IC molecules thus altering immune tolerance and leading to increased susceptibility to autoimmune diseases or to reduced immunological response against cancer cells.

A meta-analysis that included 12 studies and more than 5,000 tumor patients and an equal number of healthy controls showed a decreased cancer risk for TT homozygous individuals at polymorphism PD-1.5 (rs2227981) and, for Asian populations, a decreased risk was seen for AG individuals and an increased risk for AA, at PD-1.3 (rs11568821) (37). PD-1.5 allele frequencies and risk of low- and high-grade glioma development were examined in 156 mid-Eastern patients and significantly higher frequency of the PD1.5 C/T and T/T genotype were found in high-grade glioma compared to low-grade tumors and control individuals (38).

Some studies, described below, have examined the clinical impact of IC polymorphisms in tumor and autoimmune disease patients treated with ICi. Years ago, it was shown that SNVs in *CTLA-4* may affect the transcriptional activity of the gene as well as the interaction with CD80 and influence immune response in autoimmune diseases (39, 40). SNV of *CTLA-*4 are implicated in clinical response and survival in melanoma patients (41, 42) and, more recently, SNV-1577G > A and SNV CT60G > A were linked to a better response to Ipilimumab in a 173-patients cohort (43) and SNV-1661A > G to the onset of endocrine adverse events (44).

In NSCLC *CTLA*-*4, PD*-*1*, and *PD*-*L1* were examined in two studies that involved more than 400 patients (21, 45). Overall three *PD*-*L1* SNV (rs2282055G > T; rs4143815C > G, and rs2297136T > C) were significantly associated with a better overall response rate and improved OS and PFS when treated with chemotherapy alone or with Nivolumab as second or third line of treatment after chemotherapy.

Overall these results indicate that genetic variations in IC molecules can be utilized as conventional biomarkers predictive of response to treatment and outcome to optimize patients' treatment. This is particularly important also in view of the availability of new drugs whose efficacy and toxicity may be genetically-dependent and whose utilization requires a “personalized approach” to cancer treatment.

Studies on hematologic disorders like myelodysplastic syndrome (MDS) treated with inhibitors of DNA methyltransferases demonstrated the up-regulation of PD-1 as a consequence of the therapy (46, 47). High expression of PD-1 is a negative prognostic indicator and it has been proposed that the treatment of MDS with hypomethylating agents should be coupled with the blockade of ICs.

The analysis of several solid tumors demonstrated that epigenetic mechanisms regulate the expression of IC molecules and that methylation of PD-1 and PD-L1 promoters is associated with worse outcome (48–51).

The immunological landscape of glioma is influenced by IDH1/2 mutations; indeed, in mutated tumors PD-L1 is significantly diminished supporting the rationale of ICi treatment in IDH1/2wt patients (52). Besides DNA methylation, other epigenetic modifiers may influence IC expression in glioma. Namely, miRNAs may directly impact CTLA-4, PD-1, and PD-L1 expression through a series of miRNA like mir-155 (CTLA-4), mir-138 (CTLA-4 and PD-1), miR-424 (PD-L1 and CD80), mir-28 (PD-1), miR-34a, miR-200 miR-513, and miR-138-5p (PD-L1).

Moreover, the same or other miRNA regulate the expression of cytokines like IFN-γ or transcription factors that are positive or negative regulators of IC generating a redundant and extremely complex network. The interaction between IC molecules, and miRNA have been recently shortly reviewed (53).

## Immune checkpoint blockade in GBM: preclinical findings

Several preclinical trials conducted utilizing two immunocompetent animal models (GL261/C57Bl/6 and SMA560/VM/dk) (54) demonstrated that IC blockade utilizing ICi as single agent or in combination significantly prolongs survival at an extent that depends on the molecule, or their combination. In one study, CTLA-4 blockade alone resulted in 80% of long survivors (55) whereas in two others the percentage of long survivors was 40 and 25% (28, 56). PD-1 blockade alone resulted in 56% long survivors in one study (57) but had no effect in another study unless associated with radiotherapy (15–40% long survivors) (58). PD-L1 blockade was tested in two studies leading to 60% (57) and 25% long survivors (56). Only one study examined the effect of TIM-3 blockade with no effect on survival (59). The effect of the therapy was strongly augmented when different ICis were utilized in combination or with standard therapy. Two studies in murine models demonstrated that the combination of radiation therapy and PD-1 and/or TIM-3 exerted a strong antitumor response over the treatment with a single agent and the maximal activity (100% long survivors) was seen when PD-1 and TIM-3 inhibition were combined with stereotactic radiosurgery (58, 59). Another study described the effects of the concomitant CTLA-4/PD-L1/PD-L2 inhibition that resulted in 75% long survival (56). Finally, disabling the entire IC network (CTLA-4/PD-L1/IDO) (57) or the dual IC blockade (TIM-3/PD-1) coupled with radiosurgery (59), both resulted in the survival of 100% of the treated mice. Importantly in all these treatments it was possible to demonstrate the activation of the immune system within the tumor (cytokines production, TIL activation, etc.) and sustained anti-tumor immune response since regrowth of the tumor was not observed after tumor cell re-challenge.

Overall, these preclinical models support the rationale for disabling many components of the IC network in conjunction with standard therapies for an efficient glioma treatment. Moreover, since the concomitant utilization of several ICis could increase the risk of life threatening adverse effects, the need of identifying molecular markers predicting response and therapy-induced toxicity, as previously mentioned, appears of the utmost importance for the clinical utilization of combined IC treatment.

## Immune checkpoint blockade in GBM: clinical trials

The successful preclinical trials and the very favorable results obtained with other tumors like NSCLC and melanoma, set the basis for the utilization of ICis in many other tumors including GBM. The survey of the NIH Clinical Trials Database (https://www.clinicaltrials.gov) performed on July 2018, showed 60 registered trials; only two of them were completed (Table 1). One of the completed studies (NCT01860638) is a phase II randomized study to test the safety of the combination Bevacizumab/Lomustine as second line treatment followed by Nivolumab as third line. The primary endpoint of the study that enrolled 296 patients and ended in 2017 was OS but the results were not made available to the public. The second completed study was NCT02550249, a phase II study that enrolled 29 patients and had as primary outcome the evaluation of the expression of PD-L1 in tumor cells and lymphocytes upon treatment with Nivolumab. Also, in this case the results are not available.

**Table 1:** Clinical trials with IC inhibitors in glioma (July, 2018).

**Target**	**Clin. Trial ID**	**Molecule**	**Disease**	**Phase**	**Patients**	**Status**	**Year S/E**
CTLA-4	NCT03460782	Ipilimumab	Glioblastoma	I	?	?	2018/?
PD-1 + CTLA-4	NCT03430791	Nivolumab + Ipilimumab	Glioblastoma	II	60	Not yet recruiting	2018/2021
	NCT03233152	Ipilimumab + Nivolumab	Glioblastoma	I	6	R	2016/2019
	NCT02017717	Ipilimumab + Nivolumab + Bevacizumab	Glioblastoma	III	626	A-NR Data available (Ref. 59)	2013/2018
	NCT03367715	Ipilimumab + Nivolumab	Glioblastoma *MGMT* Unmeth	II	24	R	2018/2020
	NCT02311920	Ipilimumab + Nivolumab + TMZ	Glioblastoma Gliosarcoma	I	32	A-NR	2015/2018
	NCT03425292	Ipilimumab + Nivolumab + TMZ	Glioblastoma	I	45	R	2018/2020
	NCT03422094	Ipilimumab + Nivolumab + personalized vaccine (NeoVax)	Glioblastoma	I	30	Not yet recruiting	2018/2023
CTLA-4 + PD-L1	NCT02794883	Tremelimumab + Durvalumab	Glioblastoma	II	36	R	2016/2019
PD-1	NCT01952769	Pidilizumab	DPIG	I/II	50	A-NR	2014/2019
	NCT02359565	Pembrolizumab	DPIG and other brain tumors	I	110	R	2015/2020
	NCT02529072	Nivolumab + Dendritic cell vaccine	Glioblastoma	I	7	A-NR	2015/2017
	NCT03576612	Nivolumab + immunostimulator	Glioblastoma	I	36	A-NR	2018/2022
	NCT03557359	Nivolumab	IDHmut GB	II	37	A-NR	2018/2021
	NCT03347097	PD-1 producing pluripotent killer cells	Glioblastoma	I	40	R	2017/2018
	NCT02311582	Pembrolizumab + laser ablation	Glioma	I/II	58	R	2015/2021
	NCT02658981	Nivolumab + anti-LAG-3	Glioblastoma	I	100	R	2016/2020
	NCT02852655	Pembrolizumab	Glioblastoma	NA	35	A-NR	2016/2021
	NCT02335918	Nivolumab + Varilumab	Glioblastoma solid tumors	I/II	175	A-NR	2015/2020
	NCT02526017	Cabiralizumab + Nivolumab	Glioblastoma solid tumors	I	295	A-NR	2015/2019
	NCT03058289	INT230-6 (cytotoxic carrier, intratumor) + Nivolumab	Glioblastoma solid tumors	I/II	60	R	2017/2020
	NCT01860638	Bevacizumab + Lomustine + Nivolumab + TMZ + Radiotherapy	Glioblastoma	III	296	C - No results available	2013/2017
	NCT03014804	Dendridic cell vaccine + Nivolumab	Glioblastoma	II	30	To be started	2018/2020
	NCT03493932	Nivolumab + Anti-LAG-3	Glioblastoma	I	15	R	2018/2021
	NCT02798406	Oncolytic Adenovirus (intratumor) + Nivolumab	Nervous System Tumors	II	48	R	2016/2020
	NCT02937844	Chimeric T cells armed with PD-1 and CD28 to activate T cells and kill PD-L1+ tumor cells	Glioblastoma	I	20	R	2016/2019
	NCT03173950	Nivolumab	Brain tumors not GB	II	180	R	2017/2021
	NCT03170141	CAR-T cells	Glioblastoma	I	20	R by invitation	2017/2020
	NCT03491683	Cemiplimab + immunomodulators INO-5401 and INO-9012	Glioblastoma	I/II	52	R	2018/2021
	NCT02829931	Nivolumab + radiotherapy	Glioblastoma	I	26	S by the Company	2016/2020
	NCT02550249	Nivolumab	Glioblastoma	II	29	C - No results available	2015/2017
	NCT02648633	Nivolumab + Valproic Acid	Glioblastoma	I		WT	2016/2017
	NCT03452579	Nivolumab + Bevacizumab	Glioblastoma	II	90	R	2018/2018
	NCT02667587	Nivolumab + TMZ + radiotherapy	Glioblastoma	III	693	R	2026/2023
	NCT02617589	Nivolumab + TMZ + radiotherapy	Glioblastoma	III	550	R	2016/2019
	NCT03311542	Pembrolizumab	Glioblastoma Melanoma	?	?	?	2017/?
	NCT02313272	Pembrolizumab + Bevacizumab + radiotherapy	Glioblastoma	I	32	A-NR Data Available (Ref: 62)	2015/2019
	NCT02054806	Pembrolizumab	Glioblastoma and many solid tumors	I	477 (26 GB)	A-NR Data Available (Ref: 61)	2014/2018
PD1 + IDO	NCT03491683	Epacadostat + Nivolumab	Glioblastoma	I/II	52	R	2018/2021
PD-L1	NCT02968940	Avelumab + radiotherapy	Glioblastoma IDHmut	II	43	R	2017/2019
	NCT03291314	Avelumab + Axitinib	Glioblastoma	II	52	R	2017/2018
	NCT02866747	Durvalumab + radiotherapy	Glioblastoma	I/II	62	R	2017/2020
	NCT03341806	Avelumab + lasertherapy	Glioblastoma	I	30	R	2018/2020
	NCT02336165	Durvalumab + radiotherapy + Bevacizumab	Glioblastoma	II	159	A-NR Data Available (Ref:63)	2015/2018
	NCT03047473	Avelumab	Glioblastoma	II	30	R	2017/2019
	NCT03174197	Atezolizumab + TMZ	Glioblastoma	I/II	60	R	2017/2021
	NCT03158389	Atezolizumab + targeted therapy with various molecules	Glioblastoma	I/II	350	R	2018/2024
IDO	NCT02052648	Indoximod + radiotherapy + TMZ + Bevacizumab	Glioblastoma	I/II	160	A-NR	2014/2018
	NCT02502708	Indoximod + TMZ + radiotherapy + other cytotoxic drugs	Pediatric brain tumors	I	115	R	2015/2019
	NCT02764151	PF-06840003	Brain tumors	I	17	A-NR	2016/2018

Data taken from https://www.clinicaltrials.gov

*R, Recruiting; C, Completed; A-NR, Active Not Recruiting; AC, Accrual completed; NI, Not Indicated; WT, Withdrawn; S, Suspended; Year S/E, Year Start/End*.

The ongoing studies (mostly phase I or II) test several ICi molecules as single agents or in various combinations with standard cytotoxic molecules, targeted therapies, or other immunological therapies and are aimed, not only at determining the clinical utility of these molecules, but also at determining the safety of the treatment and are expected to be completed starting from 2019 but mostly after year 2020.

The only study with published results is NCT02017717 (CheckMate 143), a large phase III randomized trial that enrolled over 600 patients. This study was the first large trial where the effect of IC inhibitors was stringently tested. Encouraging results were initially obtained in one of the study arms where three patients showed partial response and 8 disease stabilization with the combination Nivolumab+Ipilimumab (60), however when the study was extended, this arm was closed because of the treatment failure (61).

Two other large phase III randomized trials (NCT02617589 and NCT02667587) are testing the effect of Nivolumab on MGMT methylated or unmethylated patients and the results of these studies are expected in 2019 and 2023, respectively.

Phase Ib trial NCT02054806 tested the safety and efficacy of the PD-1 inhibitor Pembrolizumab on a large series of solid tumors. In the GBM arm (26 patients), one partial response and 12 disease stabilization were observed (62).

Phase I trial NCT02313272 tested the effect of the addition of Pembrolizumab to Bevacizumab and radiotherapy. The initial results were encouraging since more than 50% of the patients at 6 months showed partial or complete response (63).

Finally, phase 2 trial NCT02336165, the PD-1 inhibitor Durvalumab was tested in combination with Bevacizumab and radiotherapy and showed partial response or disease stabilization in 60% of the patients after 6 months. Four patients remained progression free (64).

## Conclusions

Targeting ICs has revolutionized the therapeutic approach to certain tumors. There is strong hope that this therapy could be effective also for GBM patients. Indeed, the preclinical trials and the initial results obtained in some phase I/II studies suggested that ICis could offer new therapeutic options to these patients. The results of the first large phase III trials were somehow disappointing and inhibiting PD-1 could not fully restore the host immune response (61).

Nevertheless, the treatment with Nivolumab doubled the response to therapy in 8% of the patients (11.1 months vs. 5.3 with Bevacizumab) (61). Moreover, the high levels of VEGF seen in GBM are strongly immunosuppressive and this effect should be better counteracted. In this respect, targeting multiple IC pathways also in combination with cytotoxic drugs could be a winning strategy. The results of the two ongoing phase III trials and of the phase I/II trials where combination therapies are explored may provide new weapons against this rapidly and invariably deadly cancer.

## Author contributions

MR conceived the idea of this mini-review article and wrote the first draft. BB, MP, RC, AM, and MR equally participated to the final writing of the article.

### Conflict of interest statement

The authors declare that the research was conducted in the absence of any commercial or financial relationships that could be construed as a potential conflict of interest.
